# Sex-Related Differences in Gene Expression in Human Skeletal Muscle

**DOI:** 10.1371/journal.pone.0001385

**Published:** 2008-01-02

**Authors:** Stephen Welle, Rabi Tawil, Charles A. Thornton

**Affiliations:** 1 Department of Medicine, University of Rochester, Rochester, New York, United States of America; 2 Department of Neurology, University of Rochester, Rochester, New York, United States of America; Pasteur Institute, France

## Abstract

There is sexual dimorphism of skeletal muscle, the most obvious feature being the larger muscle mass of men. The molecular basis for this difference has not been clearly defined. To identify genes that might contribute to the relatively greater muscularity of men, we compared skeletal muscle gene expression profiles of 15 normal men and 15 normal women by using comprehensive oligonucleotide microarrays. Although there were sex-related differences in expression of several hundred genes, very few of the differentially expressed genes have functions that are obvious candidates for explaining the larger muscle mass of men. The men tended to have higher expression of genes encoding mitochondrial proteins, ribosomal proteins, and a few translation initiation factors. The women had >2-fold greater expression than the men (P<0.0001) of two genes that encode proteins in growth factor pathways known to be important in regulating muscle mass: growth factor receptor-bound 10 (*GRB10*) and activin A receptor IIB (*ACVR2B*). *GRB10* encodes a protein that inhibits insulin-like growth factor-1 (IGF-1) signaling. *ACVR2B* encodes a myostatin receptor. Quantitative RT-PCR confirmed higher expression of *GRB10* and *ACVR2B* genes in these women. In an independent microarray study of 10 men and 9 women with facioscapulohumeral dystrophy, women had higher expression of *GRB10* (2.7-fold, P<0.001) and *ACVR2B* (1.7-fold, P<0.03). If these sex-related differences in mRNA expression lead to reduced IGF-1 activity and increased myostatin activity, they could contribute to the sex difference in muscle size.

## Introduction

There is sexual dimorphism of skeletal muscle in overall mass, size of individual fibers, activities of several metabolic enzymes, lipid content and oxidation, relative expression of different myosin isoforms, fatigability, and expression of a number of genes [1]–[9]. Although all types of muscle fibers are larger in men, the sex difference is especially pronounced in type 2 fibers so that there is a greater ratio of type 2 fiber mass to type 1 fiber mass in men [1], [5], [10]. The sex difference in muscle mass is presumed to be mediated by higher testosterone levels in men, because of the well known anabolic effect of testosterone [11]–[13] and because estrogens and progestins do not reduce muscle mass [14]–[17]. The relative enlargement of muscle in males develops after the pubertal increase in testosterone production. After that, men require testosterone to maintain a normal muscle mass [11]–[13], [18], [19]. Testosterone, like all steroid hormones, exerts its effects by influencing gene expression. It has not been established which genes are responsible for its anabolic effects. While some effects of testosterone on gene expression might be limited to the period of rapid muscle growth after puberty, there must be some permanent effects to maintain the larger muscle mass in men.

There have been few comparisons of broad gene expression profiles in men and women. Roth et al. reported differences between men and women in the muscle expression of ∼20% of ∼1,000 transcripts that yielded reliable signals on cDNA arrays [4]. Yoshioka et al. [20] used serial analysis of gene expression (SAGE) to compare muscle gene expression in male and female mice, but SAGE is better suited for studying highly-expressed genes, such as those encoding metabolic and contractile proteins, than for studying the majority of transcripts that are expressed at lower levels. We have used comprehensive oligonucleotide arrays to study the effect of aging on expression profiles of both men and women [21], [22], but have not previously reported the sex differences. The purpose of this report is to summarize the key features of the sex-related differences in gene expression in these subjects.

## Results

We obtained muscle samples from the vastus lateralis from normal adult subjects, including 15 men and 15 women 20–75 years old. To limit variability of activity and diet prior to the biopsies, subjects were admitted to the University of Rochester General Clinical Research Center for 3 days, where they were provided a standard weight-maintaining diet and were instructed not to perform any activity more strenuous than walking. Table 1 shows their mean body composition (by dual-energy X-ray absorptiometry) and isometric knee extension strength. The men had more lean tissue mass overall (mean 41%) and in the legs (mean 48%) than the women. There did not appear to be a sex difference in physical fitness—isometric knee extension strength and maximal oxygen consumption were similar in the men and women when expressed per kg lean body mass or lean tissue mass of the legs.

**Table 1:** Mean±SEM body composition, muscle performance, and MYH gene expression patterns in 15 men and 15 women with normal muscles who donated tissue for gene expression study.

	Male	Female	P
Total body mass (kg)	76.8±2.9	64.8±1.8	0.001
Lean body mass (kg)	56.4±1.6	40.1±1.3	<0.001
Left leg lean mass (kg)	9.3±0.4	6.3±0.3	<0.001
Knee extension force/leg lean mass (N/kg)	48±3	47±2	0.81
VO_2 max_/leg lean mass (ml/min/kg)	116±7	115±6	0.91
type 1 MYH mRNA^a^	100±11	135±16	0.08
type 2a MYH mRNA^a^	100±7	70±6	0.004
type 2× MYH mRNA^a^	100±20	85±18	0.56

a % of MYH mRNA level in average male, normalized to α-actin mRNA

Table 1 also shows the expression of genes encoding types 1, 2a, and 2× myosin heavy chains (MYH). Expression of these genes was determined by RT-PCR because these highly expressed mRNAs saturated the microarray probes. Based on the fact that women have a higher ratio of type 1 fiber mass to type 2 fiber mass, the higher ratio of type 1 MYH to types 2a and 2× MYH was expected. (To avoid confusion, we did not use the official gene symbols in Table 1; types 1, 2a, and 2× fibers express the *MYH7, MYH2*, and *MYH1* genes, respectively.)

Affymetrix U133A and U133B high-density oligonucleotide arrays, containing ∼45,000 probe sets targeting all known human mRNAs and expressed sequence tags at the time of UniGene build 133, were used to examine gene expression. After elimination of probe sets that did not generate signals significantly above cross-hybridization (“mismatch”) control oligos, as well as those with signals too strong to be accurately quantified, there were ∼17,000 probe sets included in the analysis. The sex-related differences for these probe sets are available in a supporting information file (Excel File S1). The CEL files and GCRMA-normalized data for every probe set are available from the Gene Expression Omnibus (http://www.ncbi.nlm.nih.gov/geo/) via accession number GSE9676.

**Figure 1:**
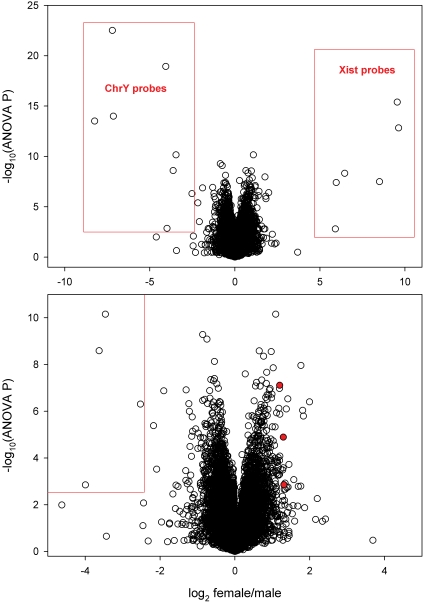
Volcano plot illustrating statistical significance of sex-related differences (shown as negative log of the ANOVA P level, so that the most significant differences are on top) in relation to magnitude of mean differences (shown as log of the female/male expression ratio, so that genes expressed at higher levels in women have positive values and those expressed at higher levels in men have negative values). The lower panel is a magnified version of part of the upper panel, excluding the very large differences for Y-chromosome genes and *XIST*, which are enclosed in red boxes in the upper panel. The three solid red circles in the lower panel represent the probe sets for the *GRB10* gene transcript.

The Significance Analysis of Microarrays (SAM) program [23], which evaluates the distribution of the t statistic with numerous random permutations of group assignments (male vs. female in this study), indicated that there were more than 3,000 sex-related differences when limiting the false discovery rate to 5%. This method did not account for age as a source of variance, so we used analysis of variance (ANOVA) as the primary method to rank genes according to the likelihood that there is a sex difference in expression after accounting for overall age effects (which have been reported previously). According to ANOVA there were more than 2,400 probe sets with a sex difference at P<0.01, more than 800 at P<0.001, and more than 300 at P<0.0001 (Figure 1). These nominal P values do not account for the fact that 17,000 comparisons were made simultaneously, but by comparison with the number of differences suggested by SAM it appears that the proportion of false discoveries at nominal P<0.01 should be very low.

As expected, the largest sex differences in signal intensity were observed with Y-chromosome genes and *XIST*, which mediates inactivation of one copy of the X chromosome in female cells [24] (Figure 1).

Table 2 lists the sex differences that stood out in the present study both in magnitude (≥2-fold) and consistency (P<0.0001 by ANOVA). Presentation of only these genes does not necessarily mean that these are the most important genes. This short list is presented to illustrate the fact that genes with various functions are differentially expressed, and that most of these genes have not been studied by researchers interested in the regulation of muscle mass. An expanded list (less stringent P and fold-difference criteria) would show the same thing.

**Table 2:** Genes with ≥2-fold differential expression in muscles of men and women at P<0.0001, excluding *XIST* and Y-chromosome genes.

gene	symbol	Fold Δ	Comment/function of gene product
**Higher expression in women**			
CDC42 binding protein kinase beta (DMPK-like)	CDC42BPB	4.0	May act as a downstream effector of Cdc42 in cytoskeletal reorganization
Ryanodine receptor 3	RYR3	3.6	Ca^++^ release
ADAMTS-like 4	ADAMTSL4	3.5	Member of ADAMTS (a disintegrin and metalloproteinase with thrombospondin motifs)-like gene family
Insulin receptor	INSR	3.4	Insulin signaling
Ubiquitously transcribed tetratricopeptide repeat, X chromosome	UTX	2.6	Escapes X inactivation
Growth factor receptor bound 10	GRB10	2.4	Inhibits insulin and IGF-1 signaling
Zinc finger protein 462	ZNF462	2.3	Transcription
Nuclear factor I/X	NFIX	2.3	Transcription
Calcium/calmodulin-dependent protein kinase (CaM kinase) II beta	CAMK2B	2.3	Kinase activity increased by exercise
Activin A receptor IIB	ACVR2B	2.3	Myostatin signaling
Calmodulin 3 (phosphorylase kinase, delta)	CALM3	2.3	Signal transduction
Forkhead box O3	FOXO3	2.2	Transcription
Bromodomain containing 4	BRD4	2.1	Chromatin binding; mitosis
Aldehyde dehydrogenase 4 family, member A1	ALDH4A1	2.0	Catalyzes the second step of the proline degradation pathway
Nuclear factor of kappa light polypeptide gene enhancer in B-cells inhibitor, alpha	NFKBIA	2.0	Signal transduction
**Higher expression in men**			
Iroquois homeobox protein 3	IRX3	4.5	Transcription
cell division cycle 37 homolog (S. cerevisiae)-like 1	CDC37L1	2.5	May function to regulate the Hsp90-mediated folding of Cdc37-dependent protein kinases into functional conformations via dimerization with Cdc37
presenilin enhancer 2 homolog (C. elegans)	PSENEN	2.3	Required for Notch pathway signaling
Dishevelled associated activator of morphogenesis 2	DAAM2	2.4	Similar to DAAM1, which has higher expression in women (1.7-fold) and is involved in Wnt/Fz signaling
tumor protein D52	TPD52	2.3	May be involved in calcium-mediated signal transduction and cell proliferation
transmembrane emp24 domain trafficking protein 2	TMED2	2.2	Protein transport
Cold shock domain protein A	CSDA	2.2	Transcription

Two of the genes expressed at higher levels in women, *GRB10* and *ACVR2B*, encode proteins that are in signaling pathways of growth factors known to regulate muscle mass. The sex difference in *GRB10* expression was especially convincing, demonstrating a 2.3- to 2.5-fold difference with three probe sets as highlighted in Figure 1 (*ACVR2B* has only one probe set). The increased insulin receptor gene expression in women could be related to the higher *GRB10* expression (see Discussion).

Because of the potential significance of *GRB10* and *ACVR2B* genes as regulators of muscle mass, we used quantitative RT-PCR as an independent method to examine their expression. The *HPRT1* transcript was selected as the reference gene on an empirical basis—*HPRT1* expression in these men (1863±111 arbitrary units, mean±SEM) and women (1881±138 arbitrary units) was similar according to the microarray data (this choice does not necessarily mean that *HPRT1* expression always is constant in muscle). According to this method, *GRB10* expression was 2.6-fold greater in the women (P<0.0001), and *ACVR2B* expression was 1.6-fold greater (P<0.01). *GRB10/HPRT1* ratios by microarray in individual subjects correlated closely with these ratios determined by RT-PCR (r = 0.91, P<0.0001). *ACVR2B/HPRT1* ratios by microarray and PCR also correlated well (r = 0.69, P<0.01).

We also examined our database of expression profiles of 10 men and 9 women with facioscapulohumeral dystrophy [25] for a sex difference in expression of *GRB10* and *ACVR2B*. The women had higher expression of *GRB10* (2.7-fold, P<0.001) and *ACVR2B* (1.7-fold, P<0.03).

Two bioinformatics resources, Gene Set Enrichment Analysis (GSEA) [26] and Expression Analysis Systematic Explorer (EASE) [27], were used to relate differentially expressed genes to functional categories. Rather than focusing on the largest differences among individual genes, GSEA integrates similar trends in expression levels among multiple genes related to one another (functionally, by co-regulation in previous experiments, by chromosomal location, or by similarity of transcription factor binding domains). According to the GSEA approach, genes expressed at higher levels in women were enriched (false discovery rate <5%) in a Chromosome 6q24 gene set. We have no explanation for this cluster. Genes expressed at higher levels in men were enriched in a “testis-expressed” set, with numerous Y-chromosome genes, and the “UVB_NHEK2_UP” gene set. The top member of the latter set in the current study was a Y-chromosome gene, and it is likely that the similarity of genes sets would not have been statistically significant without inclusion of this gene.

**Figure 2:**
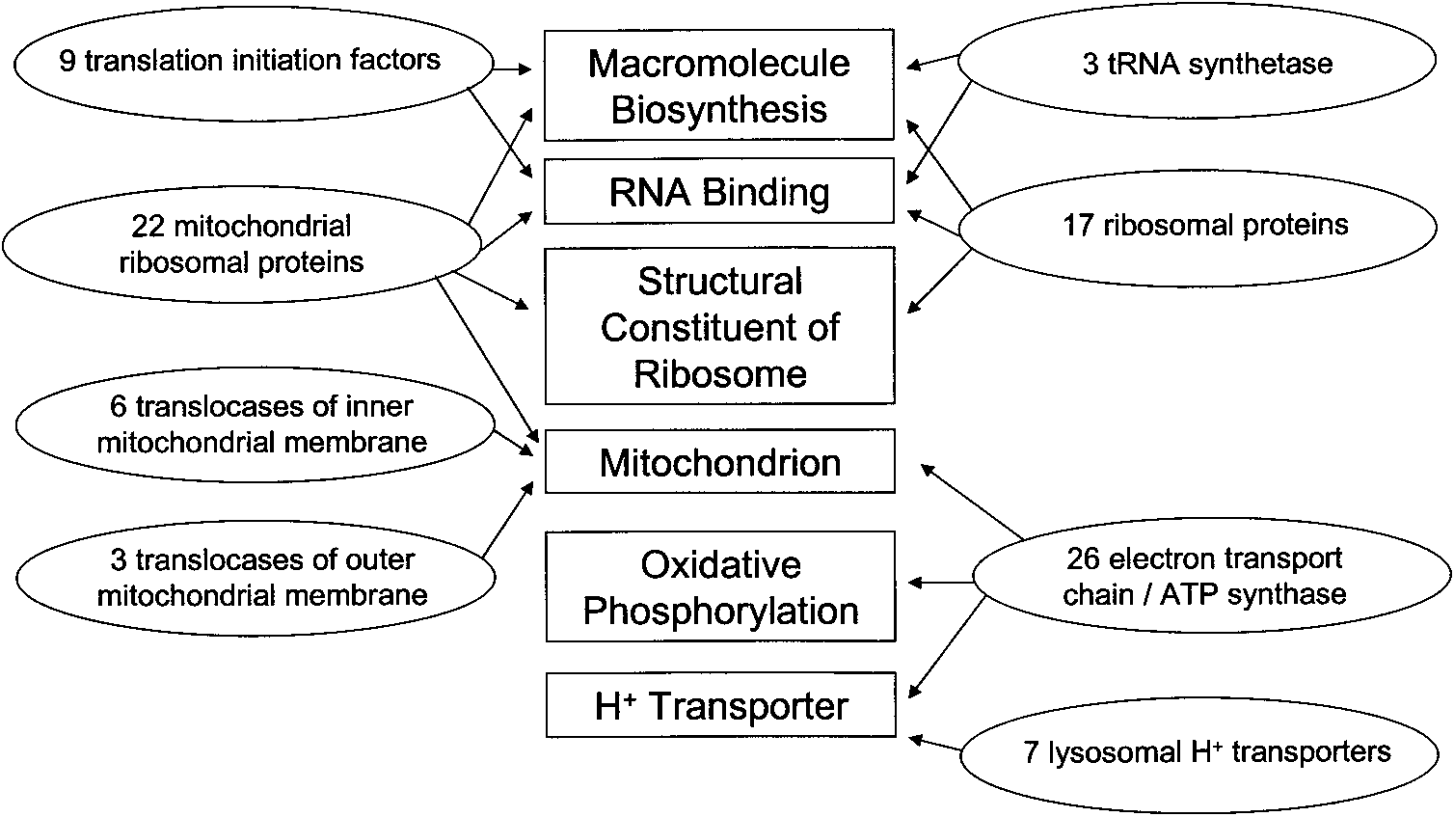
Summary of functional categories of genes expressed at higher levels in men than in women (nominal P<0.01). The percentage of genes expressed at a higher level in men that were assigned to the functional categories (rectangular boxes) was higher than would be expected by chance based on the percentage of all genes in these categories (Bonferroni P<0.05 according to EASE program). The ovals show the numbers of genes in more specific functional categories, with arrows showing how they relate to the broader categories.

EASE sorts genes according to Gene Ontology categories. This program compares a list of “significant” genes to the entire list of genes included on the array to determine if certain categories are represented more than expected by chance. Use of very strict criteria to define differential expression limits the utility of this approach, so we selected genes with sex differences at nominal P<0.01 with no fold-difference criterion. With genes expressed at higher levels in women, there were no categories enriched at P<0.05 (corrected for multiple tests by the Bonferroni method). With genes expressed at higher levels in men, there were several enriched categories (Figure 2). Genes encoding proteins involved in protein synthesis accounted for enrichment of the “macromolecule biosynthesis” category. Numerous ribosomal protein genes were the main reason for enrichment of the “RNA binding” and “structural constituent of ribosome” categories, and also were a major part of the “macromolecule biosynthesis” category. Several translation initiation factor genes also were in the biosynthesis category. The “H^+^ transporter” list was mostly genes encoding proteins of the electron transport chain and mitochondrial ATP synthase. These genes also contributed to enrichment of the “oxidative phosphorylation” and “mitochondrion” categories. Genes encoding mitochondrial ribosomal proteins contributed to enrichment of both the “biosynthesis” and “mitochondrion” categories.

## Discussion

It is well accepted that IGF-1 has an anabolic effect on muscle [28]. Although *IGF1* gene expression in muscle is sensitive to testosterone depletion or administration in men [18], [29], no sex difference in plasma IGF-1 levels or muscle *IGF1* gene expression has been observed [10]. The microarrays used in the present study also did not detect any sex difference in *IGF1* gene expression in muscle. However, IGF-1 signaling might be reduced in women because of their elevated expression of *GRB10*, which encodes a protein that interferes with IGF-1 signal transduction [30], [31]. GRB10 also interferes with insulin signaling [31], [32], in part by mediating degradation of the insulin receptor [33], but the 3-fold increase in insulin receptor gene expression in women (Table 2) could serve to minimize any negative effect on insulin sensitivity that increased *GRB10* expression might have. In contrast, there is not a sex difference in expression of the IGF-1 receptor according to the present microarray study and a previous study in which *IGFR* gene expression was determined by RT-PCR analysis [10]. Thus, the greater *GRB10* expression in women might restrain IGF-1 signaling and contribute to the sex difference in muscularity. The plausibility of this hypothesis is supported by recent studies demonstrating that *GRB10* knockout in mice was associated with increased muscularity [31], [34].

Myostatin has a major role in determining muscle size. Constitutive myostatin knockout in mice leads to a hypermuscular phenotype caused by an increased number of fibers and fiber enlargement [35]. Myostatin appears to function similarly in humans [36]. Post-developmental inhibition of myostatin activity causes muscle hypertrophy in mice without increasing the number of muscle fibers [37]–[39]. Although there is no evidence for differential expression of myostatin mRNA in men and women either from the present study or previous analyses by RT-PCR [40], [41], this does not necessarily mean that the myostatin pathway has the same level of activity in men and women. For example, myostatin protein levels in muscle are higher in female than male mice even though mRNA levels are similar in males and females [42]. Moreover, myostatin activity could differ in men and women if there is a difference in any of the proteins that process myostatin, bind to myostatin, or mediate myostatin signal transduction. Here, we report that a myostatin receptor gene, *ACVR2B*, is expressed at a higher level in women. In mice, knockout of either activin A receptor IIA (*ACVR2A*) or *ACVR2B* causes muscle hypertrophy [38]. Hemizygous knockout of *ACVR2B* accentuates muscle growth in *ACVR2A*-knockout mice, although it has no effect in mice with normal *ACVR2A* expression [38]. The microarrays indicated that the *ACVR2A* gene is expressed in human muscle, with no significant difference between men and women. Thus, understanding the functional significance of higher *ACVR2B* expression in women will require elucidation of the relative importance of ACVR2A and ACVR2B in myostatin signal transduction in humans, and whether *ACVR2B* expression is a limiting factor. It was reported recently that women with different *ACVR2B* haplotypes had different levels of quadriceps strength, although this was not observed in men, and there was no difference in leg muscle mass as assessed by DEXA [43]. It is not known whether the polymorphisms in the *ACVR2B* genes in the different haplotypes affect *ACVR2B* expression in muscle.

Because testosterone is assumed to be responsible for the greater muscle mass in men, an obvious issue is whether it regulates expression of *GRB10* or *ACVR2B* genes. Consensus androgen responsive elements [44], [45] are not near (within 5 kb) the transcription start sites of these genes. It must be emphasized that many, if not most, functional androgen responsive elements differ in several bases from the consensus motif [46]. Hence it is not currently possible to prove or rule out by computer algorithms whether testosterone regulates any particular gene. Even if there are no androgen responsive DNA elements regulating these genes directly, testosterone could influence their expression indirectly by its effects on expression of other genes. Studies of men undergoing pharmacologic inhibition of testosterone production would be the best approach to determine whether these sex differences in gene expression are mediated by testosterone.

One of the genes listed in Table 2, *FOXO3*, encodes a forkhead box transcription factor that can influence muscle catabolism. In its active non-phosphorylated state, this transcription factor stimulates expression of the ubiquitin ligase atrogin-1 (also known as MAFbx or F-box protein 32) [47]. Atrogin-1 is upregulated under conditions associated with muscle atrophy [48]. Atrogin-mediated ubiquitination of proteins increases the rate of proteasomal proteolysis. Although *FOXO3* expression was ∼2-fold higher in women than in men, there was no evidence from the microarray data for increased atrogin-1 expression in women. Moreover, there is no evidence for increased fractional muscle protein turnover in women [49]–[51], suggesting that increased muscle proteolysis is not responsible for the smaller muscle mass of women.

Most investigators using microarrays focus on the largest and most consistent differences in gene expression. However, more subtle differences in gene expression might have significant consequences, particularly if several genes involved in the same pathway are involved. We used the GSEA and EASE methods to search for sex-related differences in pathways and functional categories of genes that might not be obvious based on the largest fold differences or lowest P levels. Given the greater ratio of type 1 muscle fiber mass to type 2 mass in women, one might expect a tendency for greater expression in women of genes encoding mitochondrial proteins. However, the opposite trend was observed. There also was a tendency for the men to have higher expression of ribosomal proteins (both cytosolic and mitochondrial) and translation initiation factors. Higher expression of these genes might support an increased rate of protein synthesis in men. As mentioned above, men and women have similar fractional muscle protein breakdown rates. Therefore, men must have a higher rate of protein synthesis per muscle fiber or else they would not have a greater protein mass per fiber. (Even though synthesis per fiber is greater in men, the fractional rate is similar in men and women because protein mass per muscle fiber is greater in men.)

The GSEA program did not find enrichment in any of the Gene Ontology categories that were found by the EASE program to be more highly expressed in men. This discrepancy can be explained by the difference in the approaches of the two methods. EASE does not impose a penalty if differences opposite to the overall trend are present, whereas GSEA does because it takes into consideration all differences in both directions regardless of the statistical significance for individual genes. For example, there were 9 translation initiation factor genes expressed at a higher level in men (at nominal P<0.01), but there were 4 expressed at a higher level in women. For some other categories shown in Figure 2, the ratio of significant effects in men versus women were 22/4 (mitochondrial ribosomal proteins), 17/3 (ribosomal proteins), and 7/2 (lysosomal H^+^ transporters).

The functional significance of sex-related differences in mRNA levels depends on whether they cause differences in protein levels. Unfortunately, we do not have any more muscle tissue from these subjects to examine protein levels. We encourage others who are interested in the sexual dimorphism of muscle to examine mRNA and protein expression of *GRB10*, *ACVR2B*, or other genes that were differentially expressed in men and women in the present study.

## Methods

### Subjects

Muscle was obtained by needle biopsy, with local anesthesia, from 30 subjects with normal muscles [7 young men (21–27 yr old), 8 older men (65–75 yr old), 7 young women (20–29 yr old), 8 older women (65–71 yr old)]. Quadriceps strength, maximal oxygen consumption, and body composition measures were done as described elsewhere [21], [22]. All subjects were fully informed of risks and gave written consent for participation. The study was approved by the University of Rochester Research Subjects Review Board.

### Expression profiles

Affymetrix U133A and U133B GeneChip arrays were used to examine gene expression. Details of RNA extraction and microarray methods have been described elsewhere [21], [22]. The gene expression database was examined for differential expression in men and women after re-calculating expression scores with the GCRMA method [52] as implemented by ArrayAssist Lite software (Stratagene, Version 3.3). This method was chosen after comparing it the standard Affymetrix method, a modified version of the Affymetrix comparison analysis algorithm that was used for our analyses of age-related differences [53], and RMA [54], because it had the best performance in accounting for non-specific hybridization (expression scores in women close to zero for Y chromosome genes; in men close to zero for *XIST* gene) and had the highest correlation with quantitative RT-PCR for mean age-related differences across 8 different transcripts (r = 0.83). Probe sets with high (P>0.10) “detection P” values (Affymetrix algorithm) for more than half of the arrays from the group with higher mean expression were excluded from further analysis, which reduces the number of false positives [53], [55]. Probe sets that were saturated by very abundant transcripts also were excluded from the analysis. ANOVA for the probe sets that passed these filters was done with a Visual Basic script to calculate F ratios, which were exported to a Microsoft Excel spreadsheet for determination of nominal P values. Excel also was used for computation of mean differences between men and women. The SAM program (Version 2.21), which runs within Excel, was provided by Stanford University [23]. The GSEA program (Version 2.0) was provided by the Broad Institute [26]. The EASE program (Version 2.0) was provided by the National Institutes of Health [27].

### Quantitative RT-PCR

Expression of the MYH genes was determined with a competitive PCR method [56]. This was done prospectively, so RNA was available from all subjects for these assays. *GRB10* and *ACVR2B* transcripts were examined by quantitative real time PCR. These assays were done after the microarray analyses were completed, at which time RNA samples were available from 8 men and 7 women. *HPRT1* mRNA served as the reference transcript. RNA was treated with DNase I, then reverse transcribed by MMLV reverse transcriptase with oligo-dT as the primer. Real-time PCR quantification of cDNAs was done in triplicate reactions with the Applied Biosystems (ABI) Prism 7900HT Sequence Detection System. Primer and probe sets for TaqMan assays were purchased from ABI (for *GRB10*, assay number Hs00959287_m1; for *ACVR2B*, Hs00609603_m1; for *HPRT1*, catalog number 4333768F ). The unit of measure in these assays is the difference in cycle threshold (ΔCt) between the cDNA of interest and the reference cDNA, with an increase in ΔCt of one cycle reflecting a decrease of ∼2-fold in the *GRB10/HPRT1* or *ACVR2B/HPRT1* ratio.

## Supporting Information

Excel File S1 Sex differences for all transcripts detected in muscle. Affymetrix probe set IDs, female/male expression ratios, P values (from ANOVA), and gene names and IDs for all transcripts detected in skeletal muscle.
(3.07 MB XLS)
